# Medical Society of Tennessee

**Published:** 1835

**Authors:** 


### VII. — Medical Society of Tennessee.

*Minutes of the Proceedings of the Medical Society of Tennessee, at the Sixth Annual Meeting, held in Nashville, May 4th*, 1835.

We regret to be able to count but thirty members present at this meeting — two-thirds of whom we may presume are residents of Nashville. Why are our Tennessee brethren so backward in attendance upon the yearly meetings of their state society? The "*proceedings*" before us, in part supply an answer to this question. It appears that one object of the annual meeting is to listen to medical papers read by the members. Now we hold, that gentlemen will never neglect their business and travel from home for this purpose, when they know that all which they might hear will be published, whether they attend or not. It seems to us, that when the physicians of the different states of the West meet in societies or conventions, they should not engage in scientific investigations, but confer together on the means of uniting themselves into a profession; of giving impulse to study; of promoting objects of public benevolence; and of raising themselves, aggregately, into greater dignity and moral power, than they have yet reached in the Valley of the Mississippi. Meetings for these and similar purposes, would, we predict, be well attended, and could never fail to result in substantial benefits to the profession and society at large.

It appears that Tennessee has a law regulating the practice of physic; and that she has three Boards of Censors. Well, this is not so bad as the twenty-four Boards which lately existed in Ohio! We have, however, but little faith in the efficacy of such machinery for the elevation of the profession of medicine. It strikes us as remarkable, that the Deans of each of the Tennessee Boards should have reported, that since the last meeting of the society, no candidate had applied for examination. Does not this indicate, that the system is disapproved by the people and the profession of that state? Good physicians must be made by love of study, not fear of being rejected by boards of examiners; for, as far as our observation has extended, those who are rejected generally become favorites with the people. They are regarded as persecuted persons, and as such are sure to meet with patronage. Improved methods of study, a more protracted private pupilage, better preparatory education, and greater devotion to university opportunities, are the only safeguards against quackery; and on them alone should reliance be placed.

